# Food Delivery Service During Social Distancing: Proactively Preventing or Potentially Spreading Coronavirus Disease–2019?

**DOI:** 10.1017/dmp.2020.135

**Published:** 2020-05-05

**Authors:** Trang H. D. Nguyen, Danh C. Vu

**Affiliations:** Institute of Biotechnology and Food Technology, Industrial University of Ho Chi Minh City, Vietnam; Faculty of Technology, Van Lang University, Ho Chi Minh City, Vietnam

**Keywords:** COVID-19, delivery service, infection, pre-symptomatic

Social distancing and a shelter-in-place order are among the measures implemented to effectively prevent the spread of coronavirus disease–2019 (COVID-19).^1^ The shutdown of all nonessential services and restriction of restaurants to takeout service, in response to the social distancing measures, spark surge in food delivery service. Such a service has been touted as being a useful, convenient, and safe means to reduce the risk of exposure to infection sources of the novel coronavirus. Nevertheless, this distribution method may still pose a potential risk of spreading the disease.

**FIGURE 1:**
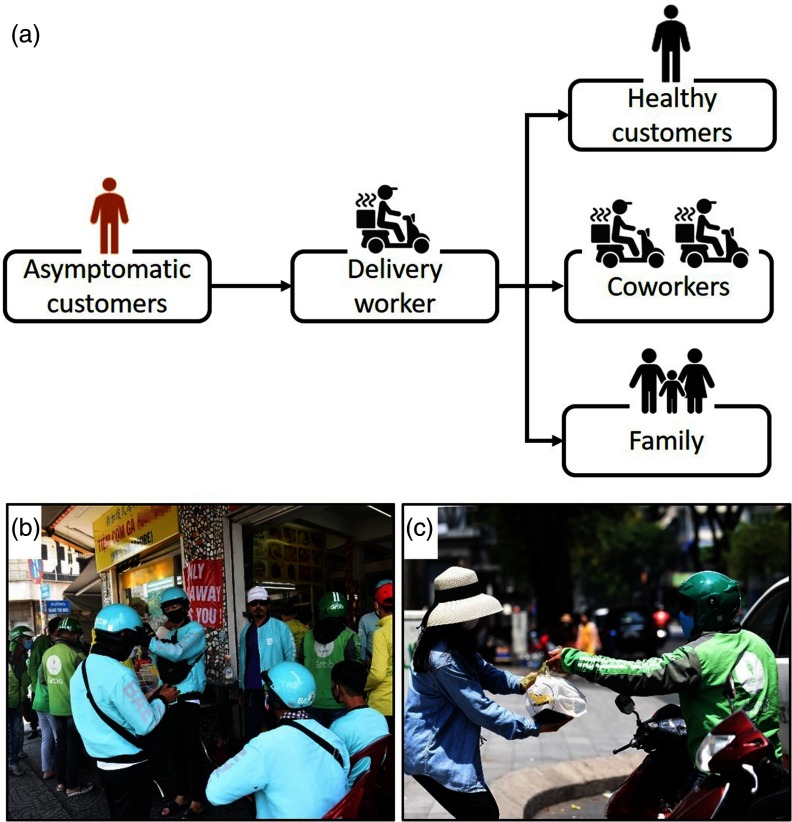
Potential spread of COVID-19 through food delivery service (a), food delivery workers at a takeout restaurant (b), and close contact with a customer (c).

Very recently, we have reported that more than 60% of the infected cases occurring in a public hospital in Hanoi, the capital of Vietnam, are linked to food delivery of mildly ill or presymptomatic nonclinical staff working at the hospital cafeteria.^2^ This has raised a concern that food delivery has a great potential of contributing to the spread of the disease.^3^ While more and more people adhere to the shelter-in-place order, delivery workers are fulfilling customer orders. This has suddenly spurred them to the frontlines of the COVID-19 pandemic.^4^ The likelihood that delivery workers (1) have direct contact with novel coronavirus–infected customers without ever experiencing symptoms and (2) may subsequently act as a presymptomatic transmitter unwittingly passing the novel coronavirus to their healthy customers, coworkers, or families should be taken into consideration (Figure 1). Evidence has shown that presymptomatic or asymptomatic transmission is 1 of the major routes by which the novel coronavirus spreads.^5^ Furthermore, 1 study indicates that presymptomatic transmission accounts for 6.4% of 157 locally acquired cases of COVID-19 in Singapore.^6^

During this time of pandemic, delivery workers are at higher risk of the novel coronavirus infection and potentially become a “spreader.” Here are practices to mitigate those risks:

1. *Contact-free delivery*. For example, in developed countries, such as the United States, Instacart offers the “Leave at my door delivery” option. In developing countries, such as Vietnam, GrabFood implements the contactless Grab transaction for which delivery workers will leave the meals at the designated position, standing 2 meters away to await customers.
2. *Strict use of new face masks, gloves, and hand sanitizers*. Delivery workers should wear new face masks and gloves, and frequently apply hand sanitizers to minimize contamination with the novel coronavirus.
3. *E-Wallet (ie, digital) or credit card payment method*. In developing countries, digital payment or credit card payment is encouraged to limit contact with delivery workers.
4. *Discarding of packaging*. Customers should discard the packaging as soon as possible and wash hands immediately after.
